# The Feeding Tube of Cyst Nematodes: Characterisation of Protein Exclusion

**DOI:** 10.1371/journal.pone.0087289

**Published:** 2014-01-28

**Authors:** Sebastian Eves-van den Akker, Catherine J. Lilley, James R. Ault, Alison E. Ashcroft, John T. Jones, Peter E. Urwin

**Affiliations:** 1 Centre for Plant Sciences, University of Leeds, Leeds, United Kingdom; 2 Cell and Molecular Sciences, The James Hutton Institute, Invergowrie, Dundee, United Kingdom; 3 The Astbury Centre for Structural Molecular Biology, University of Leeds, Leeds, United Kingdom; Griffith University, Australia

## Abstract

Plant parasitic nematodes comprise several groups; the most economically damaging of these are the sedentary endoparasites. Sedentary endoparasitic nematodes are obligate biotrophs and modify host root tissue, using a suite of effector proteins, to create a feeding site that is their sole source of nutrition. They feed by withdrawing host cell assimilate from the feeding site though a structure known as the feeding tube. The function, composition and molecular characteristics of feeding tubes are poorly characterised. It is hypothesised that the feeding tube facilitates uptake of host cell assimilate by acting as a molecular sieve. Several studies, using molecular mass as the sole indicator of protein size, have given contradictory results about the exclusion limits of the cyst nematode feeding tube. In this study we propose a method to predict protein size, based on protein database coordinates *in silico*. We tested the validity of these predictions using travelling wave ion mobility spectrometry – mass spectrometry, where predictions and measured values were within approximately 6%. We used the predictions, coupled with mass spectrometry, analytical ultracentrifugation and protein electrophoresis, to resolve previous conflicts and define the exclusion characteristics of the cyst nematode feeding tube. Heterogeneity was tested in the liquid, solid and gas phase to provide a comprehensive evaluation of three proteins of particular interest to feeding tube size exclusion, GFP, mRFP and Dual PI. The data and procedures described here could be applied to the design of plant expressed defence compounds intended for uptake into cyst nematodes. We also highlight the need to assess protein heterogeneity when creating novel fusion proteins.

## Introduction

**Figure 1:**
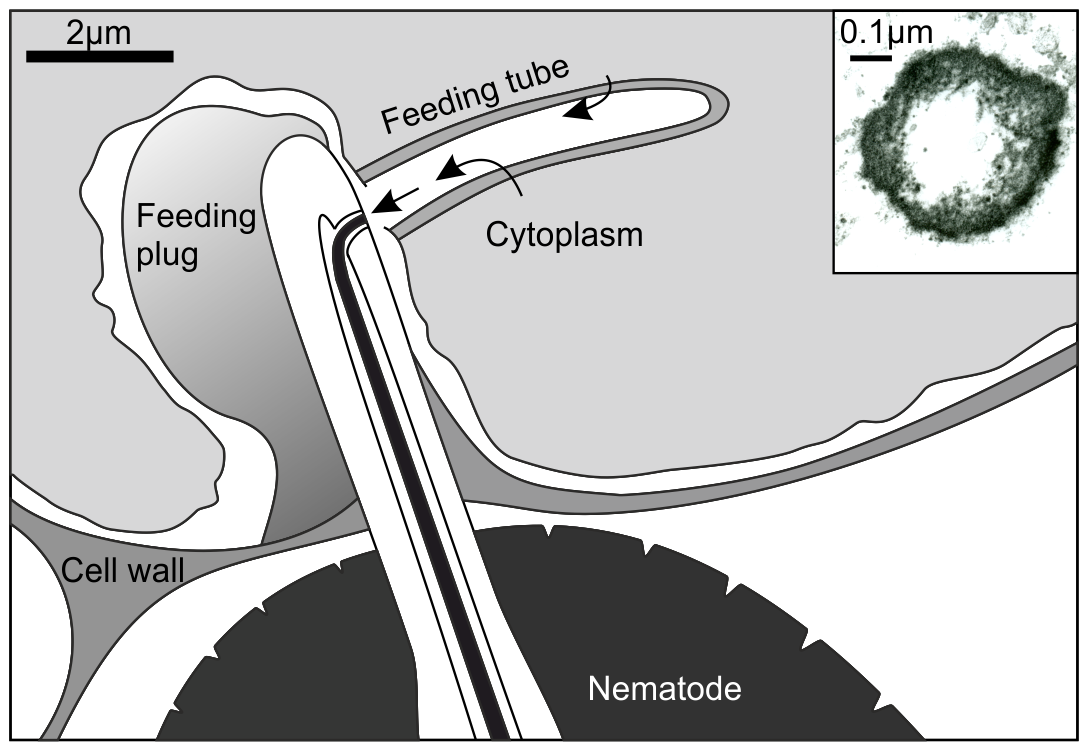
Schematic diagram of the cyst nematode feeding process. The nematode feeds by inserting the stylet through the wall of the feeding cell. A feeding tube is formed within the host cell cytoplasm, and host cell assimilate is withdrawn through the walls of the feeding tube in the direction of the arrows. Inset shows a cross section of a feeding tube induced by *Globodera pallida* in potato (*Solanum tuberosum*) and viewed under a transmission electron microscope.

Plant-parasitism has probably arisen independently in the phylum Nematoda on at least four separate occasions [1] resulting in at least one species targeting each of the world's most agronomically important crops [2]. The most economically important plant parasitic nematodes are the sedentary endoparasites. These nematodes have biotrophic interactions with their hosts and form highly specialised feeding sites within the host root tissue. The most economically important sedentary endoparasites are the root-knot nematodes and the cyst nematodes. Phylogenetic analysis has shown that the ability to induce biotrophic feeding structures has evolved independently in these two groups [1]. The cyst nematode feeding site, termed a syncytium, is formed by the fusion of multiple adjacent root cells, whereas the root-knot nematode feeding site consists of discrete “giant cells”, formed by inducing multiple rounds of mitosis in the absence of cytokinesis. Despite the differences in their ontogeny, syncytia and giant cells share a common function, and are both large multinucleate cells with proliferated endoplasmic reticulum [3], [4]. Sedentary endoparasitic nematodes feed by inserting their needle-like stylet through the host cell wall and withdrawing host cell assimilate from the feeding site [3]. Nematodes of both groups form a structure known as a feeding tube, within the feeding site. Feeding tubes are blind-ended structures, formed at the stylet orifice but within the cytoplasm of the plant cell (Figure 1).

Morphological studies show that feeding tubes differ between nematode groups. The feeding tubes of cyst and root-knot nematodes have different structures when viewed under the electron microscope and are likely to have evolved independently, suggesting that they are essential for the successful biotrophic interaction [4], [5], [6]. Although no components of feeding tubes have been identified, they are hypothesised to be of nematode origin. Different nematode species infecting the same plant produce feeding tubes characteristic of their species. Similarly, nematodes that infect different plant species produce a similar feeding tube in each host [6]. Despite morphological differences, it is likely that feeding tubes of different biotrophic nematodes share a common function. Everything the nematodes ingest must pass through the walls of the feeding tube. It has been hypothesised that the feeding tube may act as a molecular sieve to exclude large proteins/organelles, that may otherwise cause partial or total blockage of the stylet, from being taken up by the nematode [7]. Blocking of the stylet would undoubtedly be fatal as it is essential to the uptake of nutrition throughout the feeding stages of the nematode. A new feeding tube is formed before each bout of ingestion, so can be replaced if blocked. The employment of feeding tubes by the nematode may also prevent fatal damage being caused to the feeding site. Since the nematode is unable to induce further feeding sites it is essential that the feeding site is kept alive for the duration of adult development. Determining the size exclusion limit of the feeding tube has proved challenging. In previous studies, host plants expressing reporter proteins have been infected with parasitic nematodes [8], [9], [10], [11], or fluorescently labelled-dextrans have been injected directly into feeding sites [12]. Reporter molecules that are detected in the nematode digestive system can clearly pass through the feeding tube. For cyst nematode feeding tubes, there is some ambiguity in these size exclusion experiments, highlighted by a series of seemingly conflicting results. Dextrans of 20 kDa but not 40 kDa were detected within nematodes [12]. Similarly uptake of an 11 kDa single cystatin [13], and a 24.5 kDa monomeric red fluorescent protein (mRFP) [10] has been demonstrated. However, although there was no evidence for ingestion of a 22 kDa double proteinase inhibitor fusion (Dual PI) [13], the heavier 26.9 kDa green fluorescent protein (GFP) was observed to be taken up in one study [11], but not in another [8]. In experiments to date molecular mass has been used as an indicator of protein size. These apparently contradictory results suggest that a more pragmatic measure of protein size needs to be applied to feeding tube exclusion.

In recent years, more emphasis has been placed on elucidating protein structure and its link to function. Presently, the two main methods used for determining protein structure are X-ray crystallography and nuclear magnetic resonance (NMR) spectroscopy. Both give information about protein shape, but do not intrinsically tell us anything about size. Protein size can, however, be measured using ion mobility spectrometry (IMS). In conventional IMS the size of a molecule is determined by the accurate measurement of its drift time through an IMS drift tube of known length [14]. The drift tube is filled with a neutral buffer gas of known pressure. The movement of an ion through the tube, under the influence of a low electric field, is inversely proportional to its cross-sectional area and proportional to the number of charges it carries. IMS coupled with soft ionisation, such as electrospray ionisation (ESI), and mass spectrometry (MS) allow rapid determination of both a macromolecule's ‘size’ and its mass in its native state. This can be achieved in a single experiment using a much lower amount of material than required for X-ray crystallography or NMR. One type of IMS in common usage coupled with MS is travelling wave IMS (TWIMS) [15], [16]. In these devices the ion mobility separation occurs in a stacked-ring ion guide that contains the neutral buffer gas. A direct current is applied to the rings to radially confine the ions and a series of transient voltage pulses that create the travelling wave are superimposed on this. As molecules traverse the drift cell under the influence of the travelling wave, they interact with the neutral buffer gas. The frequency of these interactions, due to their ‘size’, will determine if the molecule travels along with the wave or ‘falls back’ over the wave leading to a longer drift time. The protein ‘size’ for a given mass to charge ratio is then calculated. This measure of ‘size’ is known as the temperature-dependent, rotationally-averaged, Collision Cross Sectional area (CCS). In conventional IMS this can be calculated directly from the ion's drift time. However, for TWIMS the relationship between CCS and drift time is not linear and a calibration of the device must be performed with standards of known CCS [17], [18]. It is widely accepted that there is a relationship between the CCS of a protein and its structure, as solved by NMR or X-ray crystallography. Various attempts have been made to computationally predict the CCS of proteins based on these structures [14], [19], [20], [21]. The main assumption used in these predictions is that protein conformations in the gas phase are comparable to those in crystals or solutions and, more broadly, that both of these are analogous to conformations *in vivo*. Although this assumption has been supported by a wealth of data, there are reports of protein complexes collapsing when ionised into the gas phase [22].

Of these prediction methods, the most simple is the Projection Approximation approach (PA). This calculates the CCS by averaging the area of a 2D projection of a protein over a range of viewing angles [17]. PA has been quoted to be unreliable [19], most notably because it fails to take into account the buffer gas in its predictions [23], [24]. It has been suggested that the PA approach will underestimate CCS for large concave molecules [19]. However, PA predictions have been shown to be the closest to experimental measures for predicting CCS [25], even for concave molecules such as ubiquitin [18]. In this study we introduce a new program, “RotaMol”, to predict protein size based on information from either X-ray crystallography or NMR, in a similar manner to the PA approach, using the solvent accessible surface of the protein. Having validated its agreement with experimentally determined values, we use RotaMol predictions, in conjunction with experimental methods of determining protein heterogeneity and size, to resolve conflicting data from past nematode feeding tube size exclusion experiments.

## Materials and Methods

### Computational prediction of protein size – RotaMol

#### Area measurement

The Protein Data Base (PDB) file of the protein of interest was loaded into the protein modelling program PyMol v 1.3 and used to generate a graphical representation of the protein. The 1.4 Å solvent-accessible surface was then loaded onto the protein using PyMol's built in surface function. The area of the two dimensional face, or ‘viewing angle’ that is presented to the user was recorded by counting the number of pixels which make up the protein within the PyMol viewer. The protein was then rotated by a user defined angle of rotation (θ). After each rotation a new ‘viewing angle’ was measured. The average of all the viewing angles makes up the Collision Cross Section (CCS) or ‘size’ of the protein. θ is somewhat limited by the program insomuch that 180/θ must result in an integer. Where this is not the case, the closest angle for which this is true is automatically used.

#### Measurement in pixels and conversion to Angstroms

**Figure 2:**
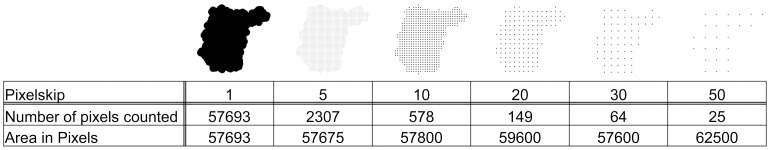
RotaMol: Varying Pixelskip and its effect on measurement accuracy. The images represent every pixel analysed at varying Pixelskips for a single viewing angle of the green fluorescent protein PDB file. At a Pixelskip of 1 every pixel is measured, at a Pixelskip of 50, 1 in every 50 pixels is measured. The number of pixels is then multiplied by the Pixelskip for X and Y.

Measuring every pixel in the protein viewer would be computationally expensive and unnecessary, as such, a new term ‘Pixelskip’ was defined. A Pixelskip of 5 will measure 1 in every 5 pixels, the pixels in between will be ignored. This builds up a coarse grained image of the protein. Figure 2 describes the loss of detail with increased Pixelskip for a single face of the PDB for GFP. For the same viewing angle, a Pixelskip of 5 will measure 2307 pixels, where a Pixelskip of 10 will measure 578 pixels. The resultant area in pixels is multiplied by the pixel skip for X and Y. For example 2307 * 5 * 5 = 57675, and similarly 578 * 10 * 10 = 57800. To convert this measure of area from pixels to Angstroms, the size of the protein viewer window in PyMol can be defined at discrete Angstrom values using the built in zoom function. Measuring the size of the window in pixels and dividing it by the size of the window in Angstroms, gives the number of pixels for one Angstrom. Full documentation, .exe and source code are available for download (http://code.google.com/p/rotamol/).

### Constructs for heterologous protein expression

Primers were designed to amplify the desired coding sequences of mRFP, GFP and Dual PI with the addition of relevant restriction enzyme sites for cloning. Each PCR product was amplified from existing plasmid templates using Phusion polymerase (New England Biolabs, Hertfordshire, UK) following the manufacturer's instructions. mRFP was amplified using an oligonucleotide primer ACACATATGATGGCCTCCTCCGAGGACGTC corresponding to the 5′ end of the coding region with addition of an NdeI restriction site, and a second primer, TGTGGATCCCTAGGCGCCGGTGGAGTGGCG, corresponding to the 3′ end of the coding region with the addition of a BamHI restriction site. GFP was amplified using an oligonucleotide primer ACAGCTAGCATGAGTAAAGGAGAAGAACTTTTC corresponding to the 5′ end of the coding region with addition of an NheI restriction site, and a second primer, TGTGGATCCCTATTTGTATAGTTCATCCATGC, corresponding to the 3′ end of the coding region with the addition of a BamHI restriction site. Dual PI was amplified from a pre-existing construct [13] using an oligonucleotide primer ACACATATGATGTCATCAGACGGAGGACC corresponding to the 5′ end of the coding region with addition of an NdeI restriction site, and a second primer, TGTGGATCCTTACTCATCATCTTCATCC, corresponding to the 3′ end of the coding region with the addition of a BamHI restriction site. The presence of an amplification product of the expected size was confirmed by agarose gel electrophoresis. 3′ A overhangs were added to the PCR product by incubating with BioTaq Red DNA polymerase (Bioline, London, UK) at 72°C for 10 minutes. Following the incubation step the PCR product was purified immediately with a Qiaquick PCR purification kit (Qiagen, Manchester, UK) following the manufacturer's instructions. Purified PCR product was cloned into the pGEM-T Easy vector (Promega, Southampton, UK) following the manufacturer's recommendations and clones confirmed by sequencing. 2 µg of pGEM-T easy plasmid containing the gene of interest was digested with the relevant restriction enzymes, the released gene fragments were gel purified (QIAquick Gel Extraction Kit; Qiagen), and ligated into digested pPET28b vector. Positive constructs, identified by restriction digestion, were transformed into the expression strain of *Escherichia coli*, BL21 DE3-RIL.

### Protein expression and quantification

Expression clones for mRFP, GFP and Dual PI were grown in 20 ml LB media (containing 100 µg/ml kanamycin and 80 µg/ml chloramphenicol) for 16 hr at 37°C (with shaking at 200 rpm). The 20 ml culture was used to inoculate 500 ml LB media, and incubated in the same conditions until A_600_ = 0.5–0.8. For both mRFP and GFP, protein expression was induced by addition of IPTG to a final concentration of 1 mM and the cultures grown for a further 3 hr at 37°C. The Dual PI expression culture was incubated for 16 hours at 16°C following induction with 1 mM IPTG. Cell pellets were collected and frozen for future protein extraction. Cell pellets were resuspended in lysis buffer (20 mM NaH_2_PO_4_, 0.3 M NaCl, 5% v/v glycerol, 10 mM imidazole and 3 µl 2-mercaptoethanol/100 ml) and incubated at 22°C for 5 minutes with 0.5 mg/ml lysozyme and 1 mM PMSF. DNA was digested by addition of MgCl_2_ to 3 mM and DNase I to a final concentration of 0.3 µg/ml. The cell lysate was cleared by centrifugation at 10,000 *g* for 20 minutes at 4°C. Clarified supernatant containing the soluble protein fraction was then purified using a HIS-Trap FF Nickel Tag Affinity (NTA) column on an ÄKTA explorer instrument (GE Healthcare, Buckinghamshire, UK) by varying concentrations of imidazole from 10 mM to 500 mM. 2 ml fractions of the eluent were collected and analysed using 12.5% SDS-PAGE to confirm expected protein molecular mass (Benchmark pre-stained Protein Ladder, Invitrogen). Pure fractions were pooled and dialysed into 150 mM NaCl, 10 mM Tris pH 7.5 using a 3 kDa membrane. Thrombin incubations to remove the His-Tag were carried out for 16 hr at room temperature at a ratio of 1∶10 w/w for thrombin to His-tagged protein. An aliquot of each digest was analysed by SDS-PAGE alongside non-digested protein to confirm that digestion was complete. The absorbance maximum of each protein sample at OD 280 nm was recorded. The amino acid sequence of each protein was analysed using ‘Protparam’ (http://web.expasy.org/protparam/ 14-10-11) to obtain an extinction coefficient. The concentration of each sample was then determined using a derivative of the Beer-Lambert Law.

### Protein analysis in solid, liquid and gas phase

Samples of each purified protein were diluted in 2X sample buffer (62.5 mM Tris-HCl pH 6.8, 25% glycerol and 1% bromophenol blue). The samples were electrophoresed in running buffer (25 mM Tris, 192 mM glycine) for 1 hour at 200 V on a 10% native polyacrylamide gel with a final concentration of 0.25 M Tris-HCl (pH 8.8). The gel was stained in Coomassie-blue for imaging. Prior to Analytical Ultra-centrifugation (AUC) samples of purified protein in PBS buffer were adjusted to an absorbance between 0.1 and 1 (280 nm) and subjected to centrifugation at 8,000 *g* for 2 minutes to remove any insoluble material. Samples were centrifuged at 200,000 rpm in an Optima XL-I Analytical Ultra centrifuge (Beckman) at 20°C. Scans were taken at 280 nm every 5 minutes for 100 scans per sample and results were analysed using SEDFIT V 12.44. Protein samples for mass spectrometry were concentrated and the buffer switched to 50 mM ammonium acetate, using Amicon Ultra 3K spin columns (Milipre, Billerica, MA, USA).

Ion mobility spectrometry-Mass spectrometry samples were analysed by Z-spray nanoelectrospray ionisation (nanoESI)MS using a quadrupole-IMS-orthogonal time-of-flight mass spectrometer (Synapt HDMS, Waters UK Ltd., Manchester, U.K.) with gold/palladium coated nanoESI tips prepared in-house. The instrument was operated in positive nanoESI-ion mobility spectrometry-TOF mode using a capillary voltage of 1.5 kV and cone voltage of 8 V. The source and desolvation temperatures were set at 80°C and 150°C, respectively. The nanoESI gas pressure was 0.1 bar, the source backing pressure was 2.4 mbar, the trap and transfer argon gas pressures were 1.8×10^−2^ mbar and the IMS cell nitrogen gas pressure was 4.9×10^−1^ mbar. The trap collision energy was 21.8 V, the transfer collision energy was 4.0 V and a trap bias of 22.8 V was used. The IMS travelling wave speed was 225 m/s and the wave height was 4.9 V. Mass calibration was performed by a separate injection of aqueous sodium iodide at a concentration of 2 µg/µl. The IMS drift cell calibration was performed by separate injection of the denatured protein standards myoglobin, cytochrome c and ubiquitin at 10 µM concentration in acetonitrile/water/formic acid (50/49/1; v/v/v). Reduced CCSs (Ω′) were calculated from published cross-sections determined using conventional ion mobility measurements (http://www.indiana.edu/~clemmer/Research/Cross%20Section%20Database-/Proteins/protein_cs.htm) and were plotted against measured drift times (tD). An allometric y = Ax^B^ fit was applied to the data. Experimental cross-sections were determined after separate infusion of the analytes and measurement of the drift time centroid for the lowest charge state ions. Data processing was performed using the MassLynx v4.1 suite of software supplied with the mass spectrometer.

## Results

### Computational protein size prediction

**Figure 3:**
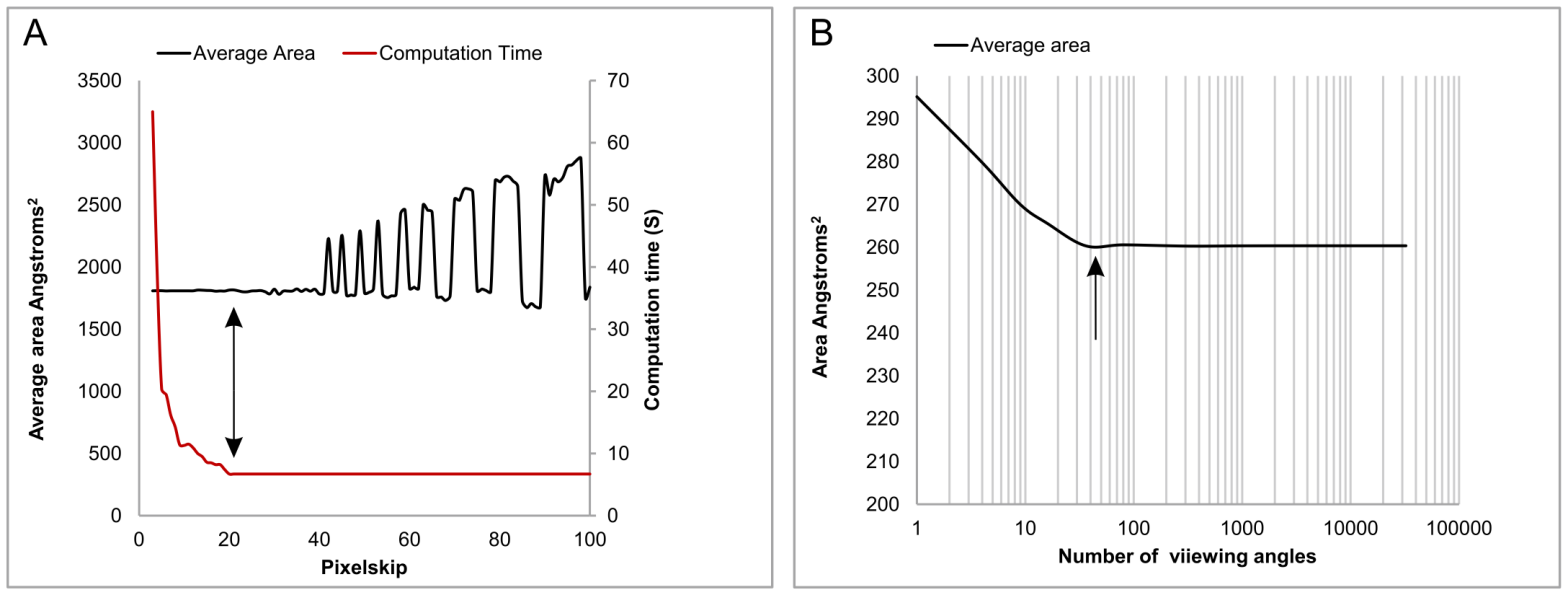
Optimum values for RotaMol parameters. (**A**) RotaMol analysis of mRFP at θ = 30° varying Pixelskip in a stepwise manner from 3 to 100. Average area (black) in Angstroms^2^ plotted against Pixelskip. Computation time (red) plotted on left axis in seconds, shows exponential increase with decreasing Pixelskip. The optimum value for Pixelskip was defined as 20, as it has the highest accuracy with the shortest computational time (arrow). (**B**) RotaMol analysis of Luteinising hormone-releasing hormone (LHRH) using a Pixelskip of 20 and varying the number of viewing angles from 1 to 32,400 (θ = 180° to 1°). The optimum value for the number of viewing angles is defined as 32 (θ = 30°) as it has no appreciable difference when compared to 32,400 viewing angles (θ = 1°).

RotaMol calculates the area of an image over a range of different viewing angles. In addition to analyte size, there are two variables that will affect the result; the resolution of the measurement (Pixelskip) and the number of viewing angles taken (inversely proportional to the angle of rotation, θ). For the Pixelskip analyses, a θ of 30° was used on the PDB model of mRFP. Pixelskip was altered in a stepwise manner for every integer between 3 and 100. Figure 3A shows the area of the protein in Angstroms^2^ (Å^2^) plotted against Pixelskip. Increasing Pixelskip from 3 to 40 resulted in no appreciable decrease in accuracy. The pattern of regular peaks and troughs above a Pixelskip of 40 is characteristic of all proteins measured (n = 20), highlighting inaccuracy of high Pixelskip values. The optimum value for Pixelskip was defined as 20, as it provided the highest accuracy for the shortest computational time.

**Figure 4:**
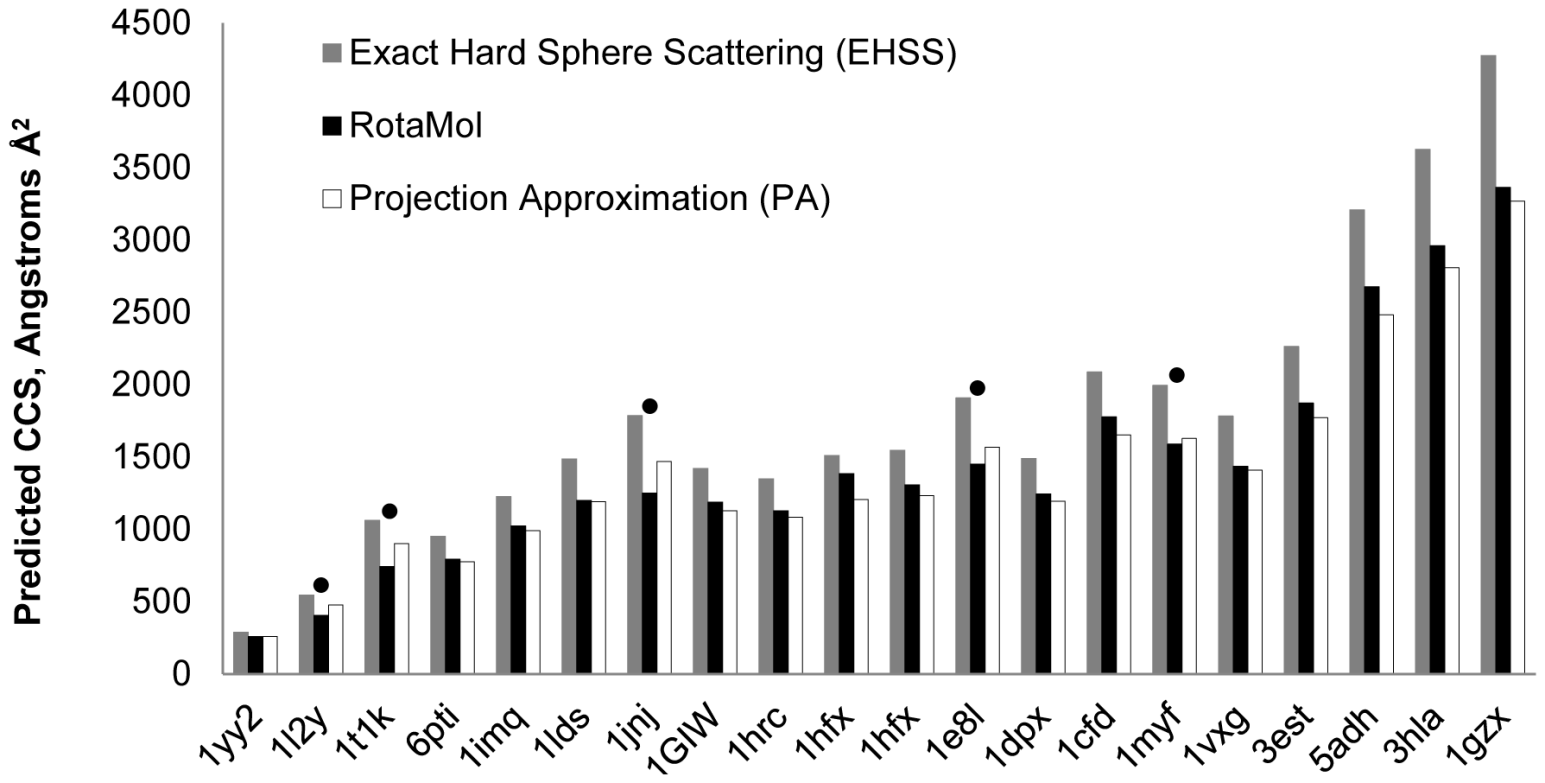
Comparison between RotaMol predictions and existing prediction methods. Size predictions shown for MOBCAL Exact Hard Sphere Scattering (EHSS) in grey bars, and Projection Approximation (PA) in white bars, compared to RotaMol, in black bars, for a range of proteins (PDB code given) from published sources. For the majority of predictions (70%) RotaMol lies between that of PA and EHSS (• indicates the cases where RotaMol predictions are not between PA and EHSS) [23], [28], [29].

For the θ analyses, a Pixelskip of 20 was used on luteinising hormone-releasing hormone (LHRH, PDB code; 1YY2). Decreasing θ results in more viewing angles being analysed. The angle of rotation θ was increased from 1° to 180°, corresponding to a change in the number of viewing angles from 32,400 to 1. Figure 3B shows the average area of LHRH plotted against the number of viewing angles used. There was no appreciable difference between the average area predicted using 32 viewing angles, and that predicted from 32,400. The optimum value for θ was therefore defined as 30°. Using these newly defined default parameters, predictions of protein CCS made by RotaMol were compared to the existing prediction methods, MOBCAL Exact Hard Sphere Scattering (EHSS) and projection approximation (PA) (Figure 4). The Trajectory Method was not included as it was not published for all proteins in the comparison. In the majority of cases (70%) the predictions made by RotaMol lay between PA and EHSS.

### Protein analysis in solid, liquid and gas phase

**Figure 5:**
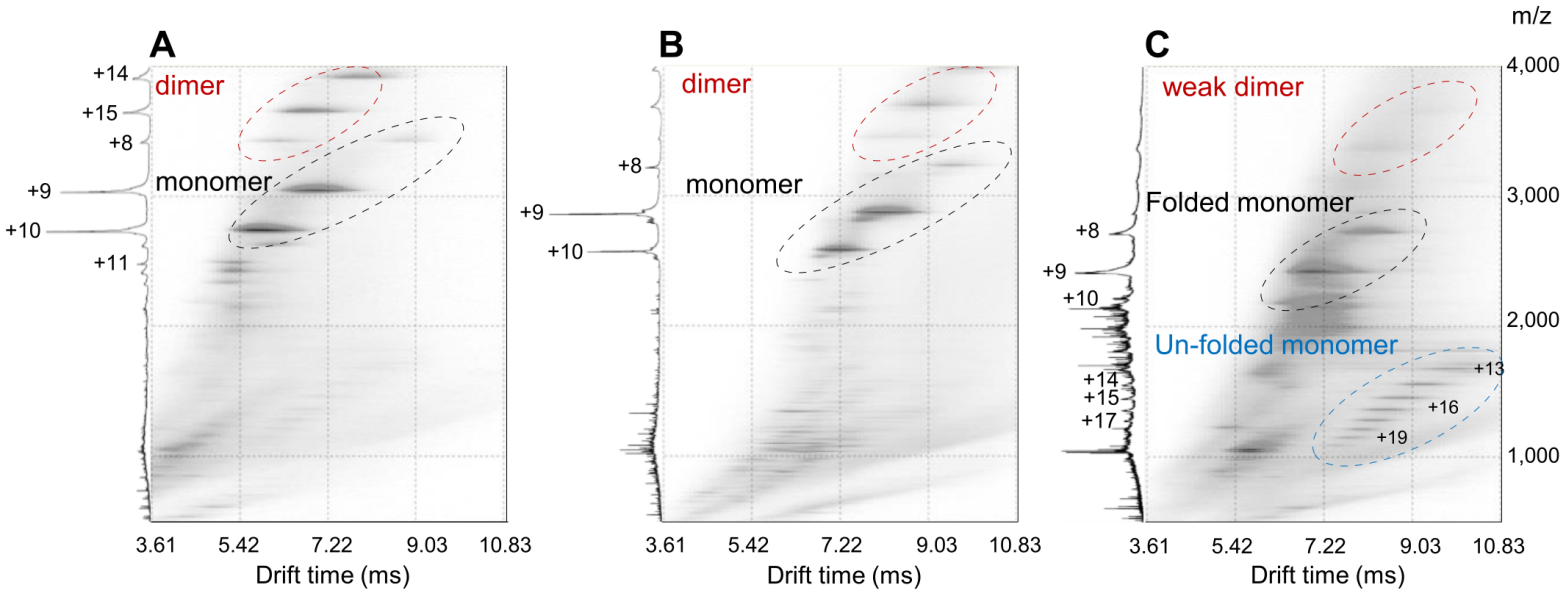
ESI-TWIMS-MS drift plots of GFP, mRFP and Dual PI. In each case, left represents mass spectrum and right represents the corresponding drift plot. (**A**) GFP drift plot shows both monomeric species of 27334 Da and a dimeric species at 54627 Da only (red). (**B**) mRFP drift plot shows a monomeric species at 25786 Da and a less intense dimeric species at 51575 Da (red). (**C**) Dual PI drift plot shows a single weak dimeric species below the measurement threshold and two monomeric species, both at 21690 Da, presumed to be folded and unfolded variants.

Having determined the optimum parameters for RotaMol, and compared its predictions to existing methods, the validity of its predictions was tested by comparison to experimentally measured CCSs. Equal quantities of purified mRFP, GFP and Dual PI in 50 mM ammonium acetate were analysed using ESI-TWIMS-MS. Figures 5A, B and C show the individual spectra and corresponding Drift plots for GFP, mRFP and Dual PI respectively. For GFP (Figure 5A), strong signals were detected for the monomeric species indicating a molecular mass of 27334 Da together with signals corresponding to a dimer at 54627 Da. mRFP was present as a monomeric form of molecular mass 25786 Da and a low abundance dimer of 51575 Da (Figure 5B). For Dual PI, a single peak indicative of a dimeric species could be detected, but in such low abundance that the mass or size could not be reported accurately. Two monomeric forms were identified corresponding to a folded and an unfolded conformer both of 21690 Da (Figure 5C). The analysis was repeated with higher concentrations of Dual PI on multiple occasions with the same consistent pattern. The measured size of the analytes was calculated by comparison to known standards, predictions were made with RotaMol (Pixelskip  = 20, θ = 30°). Table 1 shows the predicted size and measured size for both mRFP and GFP and the measured size for Dual PI. No size prediction is available for Dual PI in either conformation as there is no solved structure. Predictions for mRFP and GFP are 94.4% and 95.2% of the measured values respectively.

**Table 1:** Observed uptake of proteins by cyst nematodes correlated with their molecular mass and size.

Protein Name	Ingested by nematode	Molecular mass (Da)	Measured size (Å^2^)	Predicted size (Å^2^) (%)
mRFP	Yes	25,786	1864	1759.7 (94.4)
mRFP - dimer	unknown	51575	3126.76	N/A
GFP	Conflicting results	27,334	2007.7	1912.9 (95.2)
GFP - dimer	unknown	54627	3278.76	N/A
Dual PI - folded	No	21,690	1686	N/A
Dual PI – unfolded	No	21,690	3203.8	N/A

**Figure 6:**
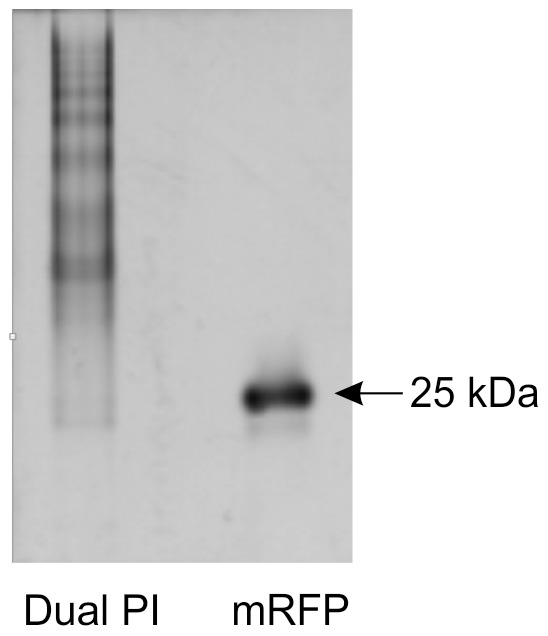
Native-PAGE comparison between Dual PI and mRFP. Dual PI migrates as multiple bands under native conditions indicating multimerisation.

**Figure 7:**
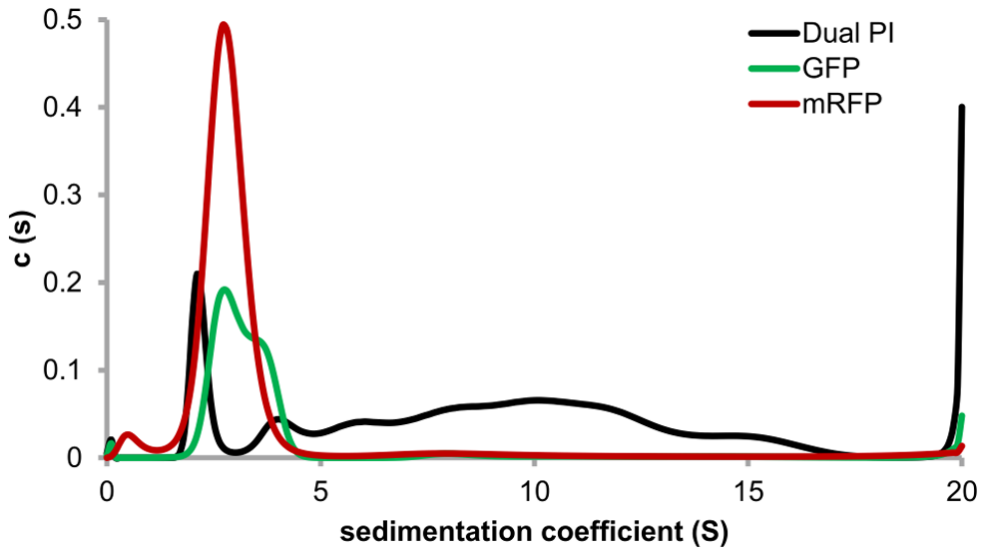
Absorbance data from analytical ultra-centrifugation for mRFP, GFP and Dual PI. For all samples, sedimentation coefficient is plotted against concentration distribution, c(s). A single monomeric species was detected for mRFP (red), both monomeric and dimeric species were detected for GFP (green). A monomeric form and large heterogeneous multimers estimated at >150 kDa were detected for Dual PI (black).

The observed heterogeneity of Dual PI was further analysed, in the solid state, using Coomassie stained native-PAGE. Under these conditions Dual PI migrated as multiple band (Figure 6). For comparison it was electrophoresed alongside mRFP, which migrated as a single monomeric species. Analytical Ultra Centrifugation (AUC) characterises the hydrodynamic properties of a protein in the liquid phase, determining the sedimentation coefficient of a protein/protein complex and providing information about hydrodynamic shape and heterogeneity without the need for interaction with a matrix or ionisation into the gas phase [26]. The AUC analysis confirmed that mRFP was present as a single monomeric species whilst both monomeric and dimeric species of GFP were present. A monomeric species was detected for Dual PI, although in low abundance. The majority of the protein was present in a large heterogeneous distribution of multimers (Figure 7), the molecular mass of which can be estimated to be a range of oligomers formed from between 4 and 12 monomers.

## Discussion

The size exclusion cut-off for cyst nematode feeding tubes has remained unclear for a number of years. In previous studies molecular mass has been used as an indicator of protein size. In this study we aimed to clarify the conflicting results between previous size exclusion experiments by assessing the size and heterogeneity of the proteins tested.

### Computational prediction of protein size

For the two measured proteins, mRFP and GFP, the CCS predictions made by RotaMol were 94.4% and 95.2% of the measured values respectively. However, these levels of agreement need to be considered in the context of other prediction methods. Of the available prediction methods, MOBCAL is the most widely used [14], [20], [27]. It has been noted on a number of occasions that the MOBCAL Projection Approximation (PA) approach has a tendency to underestimate protein size measurement [18], [19], [24], whereas the MOBCAL Exact Hard Sphere Scattering (EHSS) and MOBCAL Trajectory Method (TM) have a tendency to overestimate protein size measurement [18]. For a series of published protein size predictions and measurements, ranging from 1,000 to 64,000 Da (n = 20) [23], [28], [29], 70% of predictions by RotaMol fell between the MOBCAL PA and EHSS values (TM was not published for all proteins and so was not included in the comparison). RotaMol differs from the traditional PA approach in that it will measure any surface loaded onto the protein in PyMol; the cases described herein used the 1.4 Å solvent accessible surface. A common criticism of the PA approach is its inability to take into account electrostatic forces [24], however in place of the 1.4 Å solvent accessible surface, a projected image of external charge can be loaded onto a protein model in PyMol (Figure S1). This can then be used for downstream RotaMol analyses to give a combined measure of shape and charge. It is interesting to note that there was no difference between the average area made from 32 viewing angles of the protein and that from 32,400, despite the inherent asymmetry of the protein in question (Figure 3).

### Heterogeneity, quaternary structure, aggregation and their effect on feeding tube size exclusion

Assessing the conformations and heterogeneity of proteins used in previous size exclusion experiments has addressed the lack of correlation between molecular mass and observed passage through the feeding tube. It is widely accepted that mRFP is not excluded by any cyst nematode feeding tube. Weak dimer formation was noted in the mass spectrum (Figure 5B), however no such formation was observed in the AUC (Figure 7). ESI-MS is considerably more sensitive than AUC, and oligomers can be detected in the former but not the latter for the same sample [30]. Moreover, as the AUC analysis is performed in the liquid phase, the conformation of mRFP here is expected to be more analogous to that *in vivo*. GFP was noted to form a dimer in the mass spectrum, whilst in AUC roughly half of the GFP was present as a dimer. The GFP dimer was measured at 1.6 times larger than the GFP monomer (Table 1), assuming a similar ratio *in vivo*, detecting the remaining GFP being taken up by the nematode would be challenging. In general, it is known that protein concentration is directly proportional to aggregation under quiescent conditions [31], [32], and this is not the first report of GFP forming dimers [33], highlighting the care needed when selecting reporter proteins.

Dual PI was one of the least massive proteins tested for exclusion and yet was never detected to pass through the cyst nematode feeding tube. The mass spectrum shows weak dimer formation, but more important to note is the two different monomeric states, folded and unfolded (Figure 5). Not only does the unfolded state have a considerably larger measured size (nearly twice that of the folded), it may also play a role in aggregation [34]. The AUC revealed multiple different oligomers of Dual PI. These were in the mass range of 4–12 times that of the monomer. From this study it is unclear if the oligomers are formed from the folded protein, unfolded protein, a partially folded intermediate or a combination of all three [34], [35], [36]. These aggregates were not present in the mass spectrum, suggesting an inability to ionise some of the aggregates into the gas phase, highlighting a potential limitation of ESI-MS in assessing protein interactions in this case. AUC and Native-PAGE both highlighted the aggregates, the former suggesting that the majority of the protein (>88%) was present in a large heterogeneous distribution. This estimate of 88% is, if anything, conservative as it does not take into account any of the aggregates above a sedimentation coefficient of 20 S. When Dual PI was expressed *in planta* it was not detectable in the feeding nematode [13]. The monomeric form detected in both AUC and MS is smaller than GFP and more importantly mRFP, as such it would be expected to pass through the feeding tube. In light of our results it is likely that the Dual PI is aggregating and, as a consequence, either the aggregates are excluded by the feeding tube, or they are forming insoluble inclusions in the cytoplasm and are therefore inaccessible for uptake [36], [37]. A definitive size exclusion limit may never be found, however for homogenous proteins, the size exclusion of the cyst nematode feeding tube is likely to be at least 1,864 Å^2^. More in depth studies using a wider range of proteins may be able to resolve further the true exclusion limit. When designing fusion proteins for uptake experiments or novel compounds to target cyst nematodes, such as Dual PI, it is important that their size and/or heterogeneity is assessed before being tested *in vivo*. As we demonstrate, assessments of size can be routinely done *in silico*. Moreover, interesting work has begun to predict protein aggregation *in silico*, however at present, this is too computationally expensive for routine use [38].

### Comparing nematode feeding tube size exclusion to other biological membranes

Exclusion across other biological membranes has been previously explored and, as would be expected, the basis for exclusion depends on the membrane in question. In plasmodesmata, small channels between adjacent plant cells, hydrodynamic radius is quoted as the single determining factor of exclusion [39], [40]. This may not be the case for the cyst nematode feeding tubes. In terms of size, monomeric GFP is just 7% larger than mRFP and yet in some experiments, is excluded by the feeding tube. A sharp cut-off would be expected if the pore size of the feeding tube was uniform. This may be the case for some nematode species, such as the root-knot nematode *Meloidogyne incognita*. Electron microscope images of the feeding tube of *M. incognita* show a regular structure with discrete pores [6]. The conflicting data for the cyst nematodes suggests that pore size may follow a continuous probability distribution as would be expected from a non-uniform mesh, such as the glomerular epithelial filtration membrane [41]. Although this analogue has completely different evolutionary origins, it may share properties with the cyst nematode feeding tube. Indeed scanning electron micrographs of the cyst nematode feeding tube do not show a regular structure with discrete pores (Figure 1). The glomerular epithelial membrane is a meshwork of type IV collagens and a large number of experiments have been carried out to characterise the filtration properties of this membrane. Size exclusion of the glomerular epithelial membrane is typically defined as a sieving coefficient for a given structural unit, as opposed to a discrete limit, due to its heteroporous structure [41]. This is typical for the main manufactured form of ultrafiltration device, a depth filter. In depth filters, much of the rejection process occurs within the walls of the membrane, as opposed to at the surface in aptly named surface filters. It is interesting to note that cyst and root-knot nematodes, which biotrophic parasitism evolved independently, both create a feeding tube, each using a different type of filtration system. The cyst nematode feeding tube is more analogous to a depth filter, whereas the root-knot nematode feeding tube is more analogous to the regular structure of a surface filter. Technically, measuring sieving coefficients across the feeding tube membrane, if at all possible, will be a considerable challenge.

Size alone is not sufficient to describe filtration across the glomerular epithelial membrane. Transport of negatively charged molecules appears to be reduced relative to uncharged molecules [42]. Applying this logic to feeding tube size exclusion may explain some of the conflicting results. As previously suggested the presence of dimeric GFP will affect the outcome of size exclusion experiments, but the monomeric form itself may also pose problems. Monomeric GFP is only slightly larger than mRFP and the AUC peaks for GFP monomer and mRFP monomer lie directly on top of one another, suggesting they behave similarly in solution. The considerably larger external negative charge of monomeric GFP (Figure S1) may explain why analysis of the filtration of GFP across the feeding tube has been so difficult. The Debye-Hückel theory of charged solute interactions describes the difference in apparent size of charged molecules and pores, where a charged molecule of 29 Å will behave the same as an uncharged molecule of 37 Å (27% larger) [43]. When RotaMol is used to measure mRFP and GFP with the electrostatic fields loaded, GFP is 21% larger than mRFP, compared to 7% larger without (Table S1). A relationship between size/shape and charge may be needed to fully understand feeding tube size exclusion.

## Supporting Information

Figure S1 **Comparison of external charge on GFP and mRFP.** Electrostatic potentials, negative (red) and positive (blue), are shown for (**A**) GFP and (**B**) mRFP generated using PyMOL and the APBS tools plugin.
(TIF)

Table S1 **RotaMol predictions with and without electrostatics.**
(DOCX)

Methods S1 (DOCX)
