# Supplementary figures and images for: The Feeding Tube of Cyst Nematodes: Characterisation of Protein Exclusion
Source: PLoS One. 2014 Jan 28;9(1):e87289. doi: 10.1371/journal.pone.0087289 (PMC3905015; doi:10.1371/journal.pone.0087289)

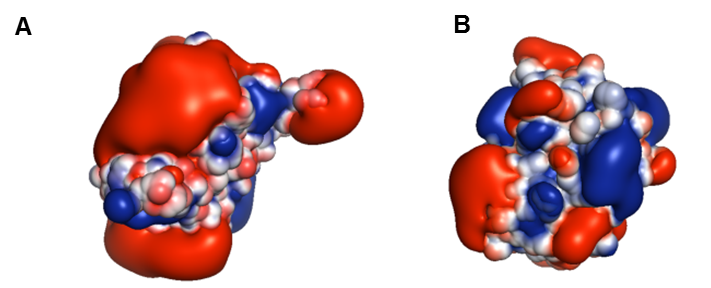

Supplement: Figure S1 — Comparison of external charge on GFP and mRFP. Electrostatic potentials, negative (red) and positive (blue), are shown for (A) GFP and (B) mRFP generated using PyMOL and the APBS tools plugin. (TIF) [file pone.0087289.s001.tif]
