# Supplementary material for: The Feeding Tube of Cyst Nematodes: Characterisation of Protein Exclusion
Source: PLoS One. 2014 Jan 28;9(1):e87289. doi: 10.1371/journal.pone.0087289 (PMC3905015; doi:10.1371/journal.pone.0087289)
Supplement: Table S1 — RotaMol predictions with and without electrostatics. (DOCX) [file pone.0087289.s002.docx]

**Table S1 RotaMol predictions with and without electrostatics**

| **Protein Name** | **Predicted size 1.4 Å surface (Å^2^)** | **Predicted size electrostatic surface (Å^2^) (% negative)** | **Percentage increase in size** |
| --- | --- | --- | --- |
| mRFP | 1759.7 | 1915.2 (53%) | 8% |
| GFP | 1912.9 | 2332.3 (65%) | 21.9% |
