# Supplementary material for: The Feeding Tube of Cyst Nematodes: Characterisation of Protein Exclusion
Source: PLoS One. 2014 Jan 28;9(1):e87289. doi: 10.1371/journal.pone.0087289 (PMC3905015; doi:10.1371/journal.pone.0087289)
Supplement: Methods S1 — (DOCX) [file pone.0087289.s003.docx]

**SUPPLEMENTARY METHODS**

**Electrostatic** **measurements**

APBS tools 2 was used in conjunction with PyMOL v1.3. For measurement of combined size/charge, both the 1.4 Å solvent accessible surface and the APBS electrostatic surface were loaded onto each protein. RotaMol was then used with a Pixelskip of 5 and an angle of rotation of 20. For percentage of external charge as in Table S1, only the electrostatic surface was loaded onto each protein. RotaMol was then used with a Pixelskip of 5 and an angle of rotation of 20.
